# Surgical resection of an intraluminal tumor in the azygos vein with an unknown primary site causing superior vena cava syndrome

**DOI:** 10.1111/1759-7714.15233

**Published:** 2024-02-05

**Authors:** Yuki Monden, Dai Une, Hidejiro Torigoe, Tetsuya Isoda, Suzuka Kamaguchi, Kenji Yoshida, Yuji Hirami, Mikizo Nakai

**Affiliations:** ^1^ Department of Cardiovascular Surgery Okayama Medical Center Okayama Japan; ^2^ Department of Thoracic Surgery Okayama Medical Center Okayama Japan; ^3^ Department of Pathology Okayama Medical Center Okayama Japan

**Keywords:** azygos vein, thoracic surgery, superior vena cava syndrome

## Abstract

Intraluminal tumor in the azygos vein is a rare disease that can cause superior vena cava (SVC) syndrome. Radiotherapy and endovascular stenting with or without chemotherapy are reported to have a high clinical success rate for the management of SVC syndrome with malignancy, but a poor survival rate. Here, we report a 69‐year‐old man who presented with swelling of the face and upper extremities, who was diagnosed with SVC syndrome caused by an intraluminal tumor in the azygos vein. Enhanced chest computed tomography revealed an intraluminal mass with a filling defect from the azygos vein to the SVC, with no extravascular extension or dissemination of the primary tumor. Surgical resection of the mass en bloc with the azygos vein and SVC reconstruction was performed. A poorly differentiated carcinoma was diagnosed on postoperative pathological evaluation. Twelve months after resection, the patient was well with no signs of recurrent disease. This case highlights that surgical resection should be considered as a treatment of choice for the management of SVC syndrome caused by an intraluminal malignancy in the azygos vein.

## Graphical abstract

We report a case of SVC syndrome caused by an intraluminal tumor in the azygos vein. Surgical resection should be considered as a treatment of choice for the management of SVC syndrome caused by an intraluminal malignancy in the azygos vein.

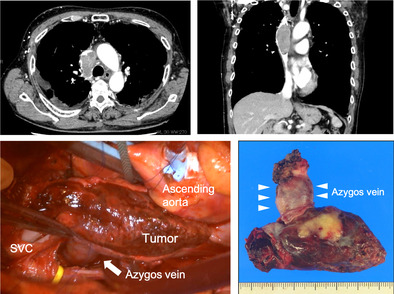

## INTRODUCTION

Superior vena cava (SVC) syndrome is caused by obstruction of the superior vena cava, most commonly by malignancy, with lung cancer being the most frequent etiology.^1^ The involvement of an intraluminal tumor in the azygos vein is rare.

Radiotherapy, endovascular stenting, and/or chemotherapy have often been used to manage malignant SVC syndrome, with surgical resection being rarely performed.^2^

Here, we report a 69‐year‐old man with SVC syndrome caused by an intraluminal mass in the azygos vein who underwent surgical resection.

## CASE REPORT

**FIGURE 1:**
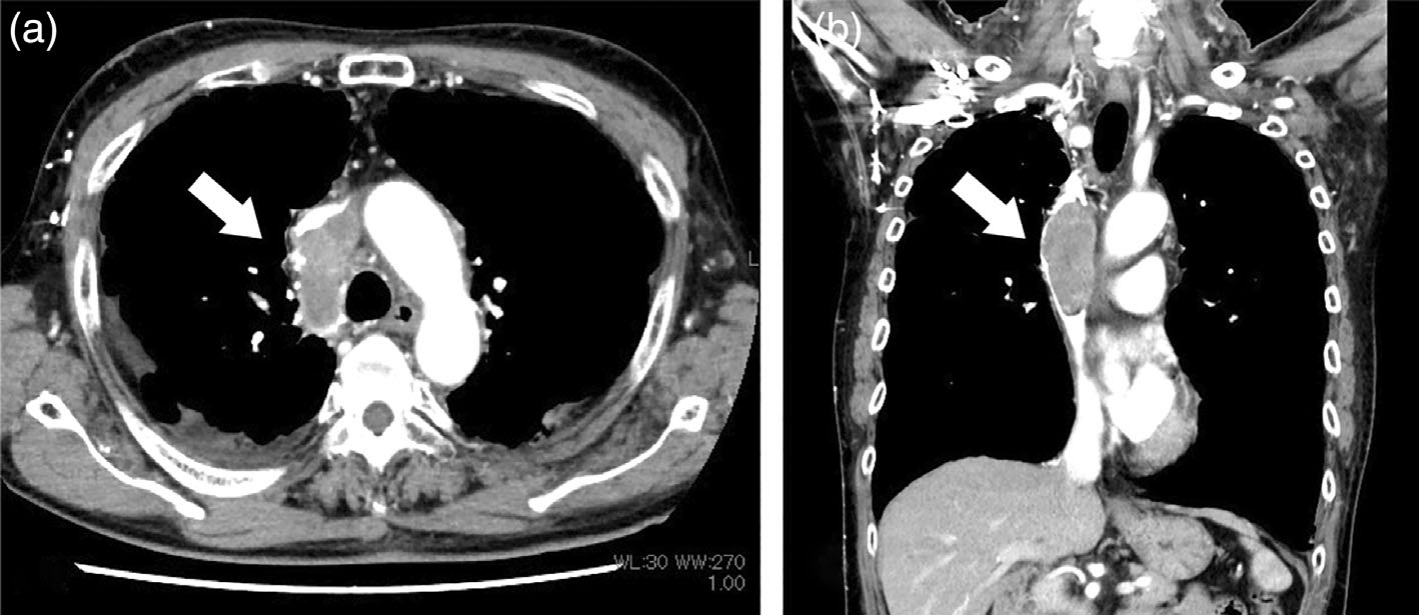
Axial (a) and coronal (b) enhanced chest computed tomography images showing a mass (arrow in each panel) in the azygos vein and the superior vena cava.

A 69‐year‐old man with a history of prostate cancer presented with swelling of the face and upper extremities. Enhanced computed tomography (CT) showed a 3 × 3 × 7 cm intraluminal mass with a filling defect extending from the azygos vein to the SVC (Figure 1a,b). No extravascular extension or dissemination from the primary tumor were evident on either CT or magnetic resonance imaging. Laboratory test results were unremarkable, including the serum prostate‐specific antigen level. We suspected leiomyosarcoma based on previous reports,^3^, ^4^ and determined that complete resection was achievable, so this was recommended. The patient declined biopsy prior to the surgery owing to the risk of hematologic dissemination to the lung.^5^

**FIGURE 2:**
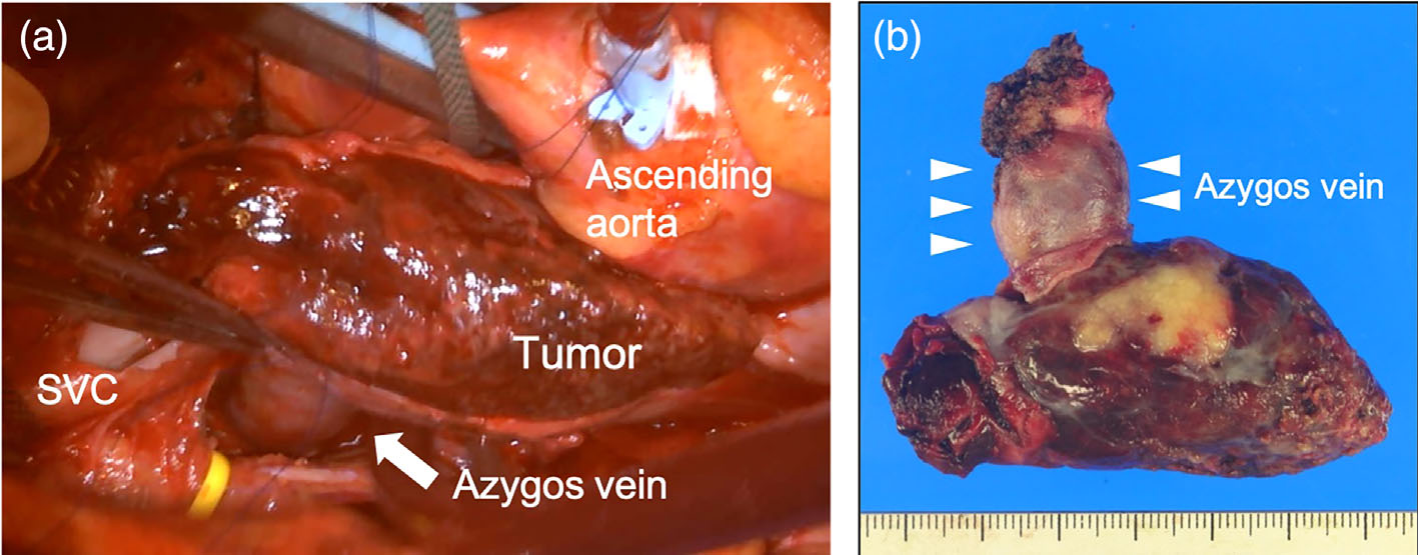
(a) Intraoperative image showing the intraluminal mass in the azygos vein and SVC after the SVC was longitudinally opened. (b) The gross appearance of the surgical specimen shows that the entire structure of the azygos vein was preserved without evident extravascular erosion. SVC, superior vena cava.

A cardiopulmonary bypass was established using a median sternotomy and fifth right intercostal thoracotomy. After the induction of cardioplegic arrest, a longitudinal incision was made on the anterior aspect of the SVC. The intraluminal mass was clearly detached from the intravascular membrane of the SVC, and resected en bloc with the azygos vein after ligation of the azygos vein distally without injury to the phrenic nerves (Figure 2). The posterior aspect of the SVC was reconstructed with autopericardium. The patient was then successfully weaned off cardiopulmonary bypass.

**FIGURE 3:**
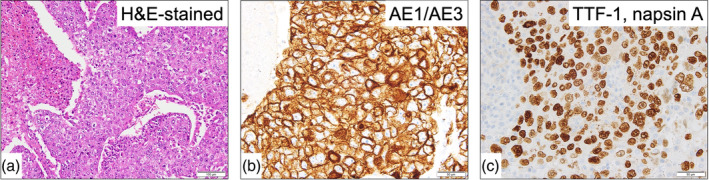
Hematoxylin and eosin (H&E)‐stained slides and immunohistochemistry (original magnification: ×40). The image shows proliferating solid nests of tumor cells with a high degree of atypia and necrosis, characteristic of poorly differentiated carcinoma (a). Tumor cells were positive for AE1/AE3 (b), TTF‐1 (nuclear staining) and napsin A (cytoplasmic staining) (c), but negative for CK14, PAX8, thyroglobulin, CDX2, NKX3.1, GATA‐3, and S100 (data not shown). AE, anion exchanger; TTF‐1, thyroid transcription factor‐1; CK14, cytokeratin 14; PAX8, paired box protein 8; CDX2, caudal‐related homeobox transcription factor 2.

Postoperatively, the facial and upper extremity swelling disappeared, and the patient recovered well with no adverse events. Histology of the specimen confirmed a poorly differentiated carcinoma, and immunohistochemical findings supported a possible carcinoma of lung origin,^6^ suggesting cancer with an unknown primary site (Figure 3). Although empirical chemotherapy was scheduled,^7^ the patient declined this owing to the risk of adverse events. At the latest follow‐up, 12 months after the resection, the patient was well with no signs of recurrent disease; however, long‐term follow‐up is necessary to monitor for recurrence.

## DISCUSSION

SVC syndrome occurs when the SVC is obstructed, typically from malignant extravascular compression by a mass in the mediastinum.^1^ Leiomyosarcoma, a mesenchymal tumor originating from smooth muscle cells of the vascular wall, is a primary malignant intravascular tumor involving the azygos vein that can cause SVC syndrome.^3^, ^4^ Intraluminal involvement of the azygos vein from renal cell carcinoma concurrently with other sites of tumor thrombus has also been reported, although not causing SVC obstruction.^8^

Metastasizing tumors, whose origin is not identified following standard diagnostic work‐up, are described as cancers of unknown primary site; they account for 3%–5% of all tumor diagnoses and are associated with intrinsic resistance to treatment and poor prognosis.^7^ Approximately 20% of patients with cancers of unknown primary site are pathologically diagnosed with poorly differentiated carcinoma, and the most common sites of involvement are the lymph nodes, lung, and bone.^7^, ^9^ To the best of our knowledge, intraluminal carcinoma of an unknown primary site presenting in the azygos vein causing SVC syndrome has not yet been reported.

Randomized studies involving patients with SVC syndrome are challenging, so data are limited.^2^ SVC syndrome management often includes chemotherapy, radiotherapy, and/or endovascular stenting.^2^, ^10^ Chemotherapy is recommended as a definitive treatment because SVC syndrome can be caused by chemosensitive malignancies; approximately 80% of patients with such malignancies achieved complete symptomatic relief, although this is lower for patients with less chemo‐sensitive malignancies such as non‐small cell lung cancer, at less than 60%.^11^

Recently, palliative radiotherapy has been reported to be an effective management with a high clinical success rate of approximately 90%.^12^, ^13^ Endovascular stenting has a similarly high clinical success rate of over 90%, and is often considered the first‐line treatment because it provides more rapid symptom resolution than radiation therapy or chemotherapy.^2^, ^13^ However, these methods were previously unsuccessful in treating patients with malignant SVC syndrome, who reported medial survival times of less than 6 months and who mostly died of cancer‐specific causes.^12^, ^13^

SVC resection and reconstruction are considered the treatment of choice for SVC invasion by lung malignancies, especially for those without lymph node involvement, with reported median survival times of up to 12 months and 5‐year survival rates of 30%–50%, compared with 5% for radiotherapy.^14^, ^15^, ^16^ Additionally, although surgeries can be technically challenging and operative mortalities of mediastinal tumors invading the SVC can be up to 10%, operative mortalities of cardiac tumor are reported to be less than 1%.^16^, ^17^ Therefore, we believe that surgery should be considered a treatment of choice for SVC syndrome with intraluminal malignancy, especially in cases when complete resection is evaluated as feasible.

In conclusion, we describe a rare case of SVC syndrome caused by intraluminal carcinoma in the azygos vein with an unknown primary site. This report highlights the need to consider surgical resection as a treatment of choice because it can achieve a better prognosis than other treatments including chemotherapy, radiotherapy, and endovascular stenting.

## AUTHOR CONTRIBUTIONS

Yuki Monden was involved in drafting the manuscript. Suzuka Kamaguchi, Kenji Yoshida, Yuji Hirami, and Mikizo Nakai were involved in data acquisition. Dai Une designed and revised the manuscript. Hidejiro Torigoe and Tetsuya Isoda revised the manuscript. All authors have read and approved the final manuscript.

## CONFLICT OF INTEREST STATEMENT

The authors declare no conflicts of interest.
